# Allochthonous matter: an important factor shaping the phytoplankton community in the Baltic Sea

**DOI:** 10.1093/plankt/fbw081

**Published:** 2016-11-26

**Authors:** J. Paczkowska, OF Rowe, L. Schlüter, C. Legrand, B. Karlson, A. Andersson

**Affiliations:** 1 Department of Ecology and Environmental Science, Umeå University, SE-901 87Umeå, Sweden; 2 Umeå Marine Science Centre, SE-905 71Hörnefors, Sweden; 3 Department of Food and Environmental Sciences, Division of Microbiology and Biotechnology, Viikki Biocenter 1, University of Helsinki, Helsinki, Finland; 4 DHI, Environment and Toxicology, Agern Allé 5, 2970Hørsholmc, Denmark; 5 Center of Ecology and Evolution in Microbial Model Systems, EEMiS, Department of Biology and Environmental Sciences, Linnaeus University, SE-391 82Kalmar, Sweden; 6 Swedish Meteorological and Hydrological Institute, Oceanography, SE-426 71Västra Frölunda, Sweden

**Keywords:** phytoplankton, allochthonous dissolved organic matter, humic substances, nitrogen, phosphorus, structuring factors

## Abstract

It is well-known that nutrients shape phytoplankton communities in marine systems, but in coastal waters allochthonous dissolved organic matter (ADOM) may also be of central importance. We studied how humic substances (proxy of ADOM) and other variables influenced the nutritional strategies, size structure and pigment content of the phytoplankton community along a south–north gradient in the Baltic Sea. During the summer, the proportion of mixotrophs increased gradually from the phosphorus-rich south to the ADOM-rich north, probably due to ADOM-fueled microbes. The opposite trend was observed for autotrophs. The chlorophyll *a* (Chl *a*): carbon (C) ratio increased while the levels of photoprotective pigments decreased from south to north, indicating adaptation to the darker humic-rich water in the north. Picocyanobacteria dominated in phosphorus-rich areas while nanoplankton increased in ADOM-rich areas. During the winter–spring the phytoplankton biomass and concentrations of photoprotective pigments were low, and no trends with respect to autotrophs and mixotrophs were observed. Microplankton was the dominant size group in the entire study area. We conclude that changes in the size structure of the phytoplankton community, the Chl *a*:C ratio and the concentrations of photoprotective pigments are indicative of changes in ADOM, a factor of particular importance in a changing climate.

## INTRODUCTION

Phytoplankton communities are shaped by bottom-up and top-down factors (Chisholm, 1992; Kiørboe, 1993; Mousing *et al*., 2014), and their community properties are in turn important for aquatic food web functioning (Havens, 1998; Dahlgren *et al*., 2010). Small phytoplankton cells possess advantages over larger cells for resource acquisition, growth rate and photosynthetic rate under low-nutrient and light-limiting conditions (Grover, 1989; Raven, 1998). Dominance of small phytoplankton results in more internal trophic levels in the food web and thus reduced food web efficiency (Legendre and Rassoulzadegan, 1995). Larger phytoplankton cells dominate in turbulent and nutrient-rich environments, inducing shorter food chains with higher food web efficiency (Legendre and Rassoulzadegan, 1995). Because of the interrelationship between nutrients and temperature in aquatic systems, changes in the size structure of the phytoplankton community are often caused by concurrent variations of these factors (Agawin *et al*., 2000; Mousing *et al*., 2014).

Due to the interaction with the heterotrophic microbial food web, the size distribution and function of the phytoplankton community is not only influenced by factors like nutrient availability and temperature. In coastal areas and semi-enclosed seas like the Baltic Sea, the influence of allochthonous dissolved organic matter (ADOM) can be strong, fueling the heterotrophic microbial food web (Andersson *et al*., 2015; Figueroa *et al*., 2016). Under such conditions mixotrophic phytoplankton may also be enhanced, since they feed, e.g. on heterotrophic bacteria (Andersson *et al*., 1989). Changes in ADOM input can be influenced both by climate-related factors, such as temperature, precipitation and hydrology or by alterations in acidity or land-use activity (Evans *et al*., 2006; Erlandsson *et al*., 2008). Climate change projections indicate that both the temperature and precipitation will increase in northern Europe. The river inflow of freshwater to the Baltic Sea will thus increase, causing decreased salinity and increased concentrations of ADOM (Meier *et al*., 2012; Andersson *et al*., 2015). How an increase in ADOM will affect phytoplankton structure in the Baltic Sea is poorly understood.

The brownish color of ADOM attenuates light in the water and hampers primary production (Thrane *et al*., 2014; Seekell *et al*., 2015). It also leads to changes in the cellular concentration of pigments in phytoplankton (Falkowski and Raven, 2007). Phytoplankton respond to decreasing light by increasing their concentrations of chlorophyll *a* (Chl *a*) and accessory photosynthetic pigment content in order to harvest as much light as possible (MacIntyre *et al.,* 2002; Behrenfeld *et al*., 2005). Under poorer light conditions smaller cell sizes with large *S*/*V* ratios are also beneficial, because the light-harvesting pigments are positioned closer to the cell surface and are more evenly distributed in the cells (Raven, 1998; Kirk, 2011). In high light conditions the photoprotective pigments in phytoplankton will increase and larger cells become promoted. Furthermore, the taxonomic composition and nutrient availability influence pigment concentrations, e.g. as nitrogen is essential in the synthesis of pigment–proteins complexes (Dubinsky and Stambler, 2009; Edwards *et al*., 2015; Spilling *et al*., 2015). Even though such physiological acclimatization's are known from laboratory studies, it is not known whether similar responses occur in natural systems.

The objective of this study was to elucidate how bottom-up factors, such as ADOM, nitrogen, phosphorus and temperature, influence properties in the phytoplankton community in the semi-enclosed Baltic Sea. Samples were collected along a south–north gradient during a summer and a winter–spring campaign, to elucidate factors governing the general size structure, nutritional strategy and pigment content of the phytoplankton community. The following hypotheses were tested: (i) high phosphorus and nitrogen concentrations lead to large phytoplankton cell sizes, (ii) high ADOM concentrations promote mixotrophs and drive phytoplankton to increase their chlorophyll *a* to carbon ratio (Chl *a*:C) and (iii) low ADOM drives phytoplankton to increase their levels of photoprotective pigments. This study contributes to the general understanding of marine ecosystem system function as well as consequences of climate change, since in the Baltic Sea the ADOM and nutrient concentrations are expected to change during the course of the next 100 years (Andersson *et al*., 2015).

## METHOD

### Field sampling

**Fig. 1.**
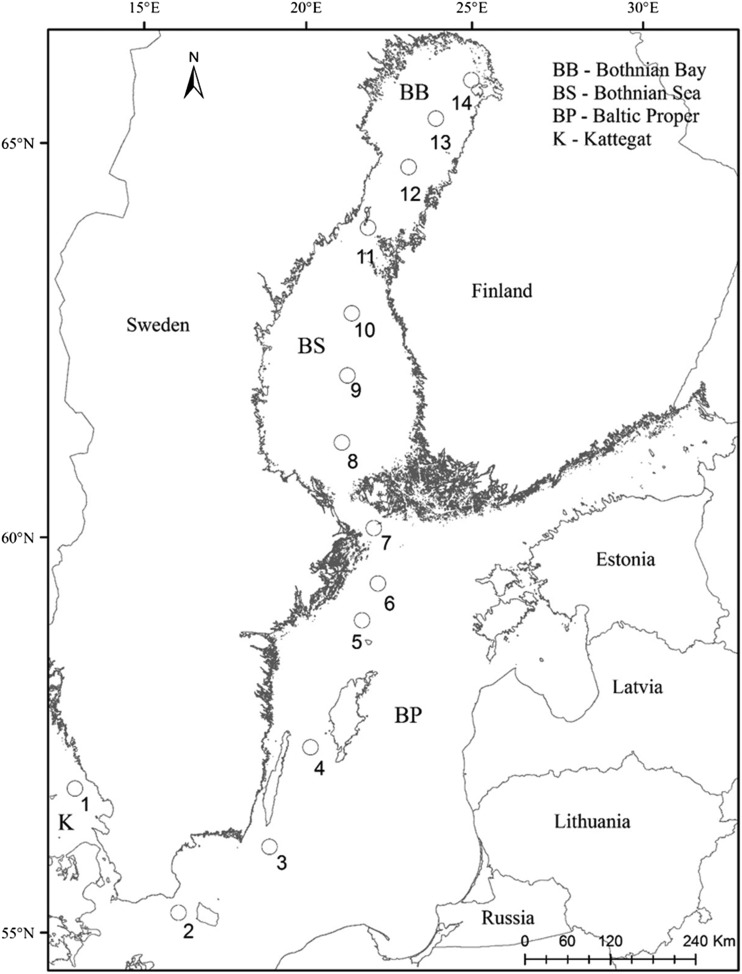
Sampling locations along a south–north transect in the Baltic Sea.

Spatial and temporal variation in the phytoplankton community and physicochemical variables were investigated during a late summer (23–25 August 2011) and a winter–spring (16–19 March 2012) sampling campaign. Fourteen stations were sampled along a south–north gradient in the Baltic Sea (Fig. 1, Table S1, Supplementary material online). Ice cover was present in the Bothnian Bay in March, and reached 50 cm in the northernmost part of the basin. Samples were collected from a Ferry Box system, installed on a cargo ship (TransPaper) traveling between Gothenburg (Sweden) and Kemi (Finland). Temperature and salinity were measured using an SBE Temp 38/SBE TSG 45 sensor. Water samples were handled and preserved on board within 2 h of collection.

### Chemical analyses

For the analysis of total phosphorus (Tot P) and total nitrogen (Tot N), 50 ml of unfiltered water were frozen (**–**20°C) in Falcon tubes and stored until analysis. Measurements were carried out using a Braan and Luebbe TRAACS 800 autoanalyzer, following standard analytical methods (Grasshoff *et al*., 1983). Tot N and Tot P were considered to indicate the carrying capacity of the system, and also to reflect the nutrients available to the phytoplankton. This assumption is based on results of other studies performed in the Baltic Sea, where Tot N and Tot P concentrations were shown to be positively correlated with inorganic nutrients (data not shown). Furthermore, nutrient turnover has been shown to be relatively rapid in aquatic systems: Kress *et al*. (2005) showed that in the Mediterranean Sea the nutrient turnover time was <1 h when nutrient limitation prevailed, while when nutrients were replete the nutrient turnover time increased to 3–4 days. In the Baltic Proper, Tot P turnover related to the phytoplankton production was estimated to be 3.2 days (Håkansson and Bryhn, 2008).

Humic substances (HSs) were analyzed and used as a proxy for ADOM. Samples of 100 ml were kept in amber-colored glass bottles in the dark at 4°C and measured with a Perkin Elmer LS 30 fluorometer at 350/450 excitation/emission wavelengths. Calibration standards were prepared from quinine dihydrogen sulfate dihydrate in 0.05 M sulfuric acid (Hoge *et al*., 1993; Wedborg *et al*., 1994). Sulfuric acid (0.05 M) was used as a blank.

### Pigments

Samples (200–800 ml) were filtered onto 25 mm GF/F filters, frozen in liquid nitrogen and stored at –80°C. To extract the pigments, filters were placed in 3 ml 95% acetone containing vitamin E as an internal standard, sonicated in an ice bath for 15 min and extracted at 4°C overnight in the dark. After extraction, the samples were filtered through a 0.2 µm Teflon syringe filter into vials, injected into a Schimadzu LC-10ADVP (High Performance Liquid Chromatography: HPLC) and analyzed according to van Heukelem and Thomas (van Heukelem and Thomas, 2001), with slight modifications for the local conditions (Schlüter *et al*., 2014) to identify and quantify the phytoplankton pigments. The photoprotective pigment index (PI), a measure of the physiological state of the phytoplankton community, was calculated according to Moreno (Moreno *et al*., 2012):

PI=(diadinoxanthin+diatoxanthin+zeaxanthin)/Chla

### Phytoplankton

For the analysis of nanoplankton and microplankton, two 50 ml samples were preserved with 2% acidic Lugol's solution, settled in sedimentation chambers for 24–48 h and counted at ×100 (microplankton) and ×400 (nanoplankton) magnification using a Leica DM IRB inverted microscope (Utermöhl, 1958).

The nutritional strategy of the phytoplankton (autotrophic, heterotrophic or mixotrophic) was classified according to Olenina (Olenina *et al*., 2006). To study the size structure, the phytoplankton cells were divided into four size categories: <2 µm (picoplankton), 2–10 µm (ultraplankton), 10–20 µm (nanoplankton) and >20 µm (microplankton), based on measurements of the longest cell axis. Tightly connected cells of filamentous cyanobacteria were grouped into the >20 µm fraction.

Samples for the analysis of picocyanobacteria were fixed with glutaraldehyde (2% final concentration) and filtered (2–5 ml) onto 0.6 µm black polycarbonate filters. The samples were counted using an epifluorescence microscope (Nikon Eclipse TE 2000-U) at ×1000 magnification, using green excitation light (510–560 nm, emission wavelength > 590 nm). At least 200 cells per sample, in 20 randomly positioned view fields, were counted.

The phytoplankton biovolume was calculated according to the size and geometry of the cells (Olenina *et al*., 2006). Cell carbon was calculated from the biovolume (Menden–Deuer and Lessard, 2000), and carbon biomass concentrations from cell abundance and cell carbon. The Chl *a*:C ratio indicated the chlorophyll *a* content in the phytoplankton cells, and was calculated by dividing the Chl *a* concentrations with the phytoplankton carbon biomass concentrations.

### Statistical analyses

Physicochemical and biological variables were tested for normality using the Shapiro–Wilk test and homogeneity by Levene's test. Variables that did not fulfill for a normal distribution were either logarithmically transformed or arcsin–square root transformed (contribution of mixotrophs, autotrophs to the total biomass and Chl *a*:C ratio, photoprotective PI). The Student's *t*-test was used to test differences in phytoplankton total biomass, phytoplankton size structure, chlorophyll *a* and physicochemical variables between the summer and winter–spring sampling seasons. To explore relationships between physicochemical and biological variables, Pearson's rank correlation was performed. Principal component analysis (PCA) was used to show the main patterns of phytoplankton size structure, nutritional strategies and pigment content in relation to potentially explanatory variables during the summer and winter–spring samplings. Non-metric multidimensional scaling (NMDS) plot based on Bray–Curtis similarity matrix was performed to visualize differences in phytoplankton composition between stations and seasons. To test similarities in phytoplankton composition between sampling periods, ANOSIM analysis was conducted. Station 1 was excluded from analyses because the values differed substantially from the other stations as a result of the strong influence of the North Sea. Data analyses were performed in SPSS Statistics 23, Primer 6 and Canoco 5.

## RESULTS

**Fig. 2.**
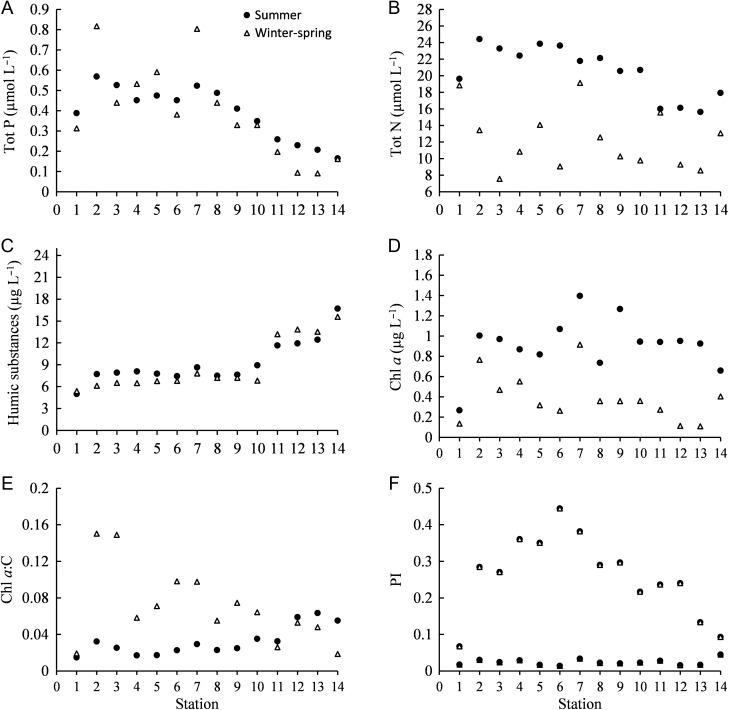
Spatial distribution of (**A**) total phosphorus (Tot P), (**B**) total nitrogen (Tot N), (**C**) humic substances, (**D**) chlorophyll *a* (Chl *a*) content, (**E**) Chl *a*:carbon (C) ratio and (**F**) photoprotective pigment index (PI) during summer and winter–spring sampling periods.

The physicochemical variables in general showed spatial and temporal variations (Table S1, supplementary material online). The salinity was 18–20 in the Kattegat and decreased to 2–3 in the Bothnian Bay. The average temperature was 17 and 2°C in the summer and winter–spring, respectively. Tot P showed a decreasing trend from south to north during both summer and winter–spring, while Tot N showed a similar spatial pattern only during the summer (Fig. 2A and B). The Tot P concentrations were relatively similar during both periods (Student's *t*-test: *t* = 0.056, df = 24, *P* = 0.956), while the Tot N concentrations were higher during summer (Student's *t*-test: *t* = 6.786, df = 24, *P* < 0.001). The concentrations of HSs increased from south to north with highest values in the Bothnian Bay (Table S, supplementary material online, Fig. 2C). Overall HSs did not differ between seasons (Student's *t*-test: *t* = 0.645, df = 24, *P* = 0.525). Pearson correlation analysis showed that many of the physicochemical variables were correlated to each other (Table S2). For example, HSs were negatively correlated to Tot P, salinity and temperature during both the summer and the winter–spring samplings.

### Distribution of Chl *a*

The Chl *a* concentrations were higher in summer than in winter–spring (Fig. 2D, Student's *t*-test: *t* = 6.668, df = 24, *P* < 0.001). During summer the concentrations were ~1 µg L^−1^ at most of the stations. In winter–spring there was an overall decreasing trend from the southern Baltic Proper (0.8 µg L^−1^) to the northern Bothnian Bay (0.1 µg L^−1^). However, it is notable that during both seasons highest concentrations were observed at the entrance of the Gulf of Bothnia, while the lowest occurred in the Kattegat.

### The Chl *a*:C ratio and PI

The Chl *a*:C ratio increased from south to north during the summer, while the opposite trend was observed during the winter–spring (Fig. 2E). However, the lowest Chl *a*:C ratio was observed at station 1 (Kattegat), during both seasons. In the summer, the Chl *a*:C ratio showed a positive correlation with HSs and a negative correlation with salinity, temperature, Tot N and Tot P (Table I). In the winter–spring, the Chl *a*:C ratio showed a negative correlation with HSs and a positive correlation with temperature, salinity and Tot P (Table I). Overall, our data showed a 10-fold difference in the Chl *a*:C ratio.

**Table I:** Pearson correlation coefficients between total phytoplankton biomass, different size groups (<2, 2–10, 10–20 and >20 µm), autotrophs (AU), mixotrophs (MX), chlorophyll *a* (Chl *a*), chlorophyll *a*: carbon ratio (Chl *a*:C), photoprotective pigment index (PI) and physicochemical variables [salinity, temperature (Temp), total nitrogen (Tot N), total phosphorus (Tot P), humic substances (HSs)].

Season	Variables	Total biomass	<2 µm	2–10 µm	10–20 µm	>20 µm	AU	MX	Chl *a*	Chl *a*:C	PI
Summer	Salinity	0.839**	0.865**	−0.892**	−0.801**	0.667*	0.844**	−0.386	0.321	−0.827**	0.807**
	Temp	0.666*	0.627*	−0.345	−0.630*	0.630*	0.661*	0.059	0.313	−0.601*	0.806**
	Tot N	0.745**	0.737**	−0.818**	−0.682*	0.635*	0.747**	−0.278	0.156	−0.801**	0.692**
	Tot P	0.827**	0.846**	−0.932**	−0.849**	0.699**	0.830**	−0.259	0.378	−0.804**	0.791**
	HS	−0.878**	−0.879**	0.856**	0.902**	−0.783**	−0.881**	0.253	−0.397	0.839**	−0.845**
Winter**–**spring	Salinity	−0.132	0.343	−0.002	0.009	−0.318	−0.140	0.155	0.615*	0.798**	−0.075
	Temp	0.135	0.027	0.344	0.474	−0.006	0.130	−0.031	0.588*	0.559*	0.191
	Tot N	0.658*	0.576*	−0.165	−0.026	0.624*	0.664*	−0.112	0.553*	−0.189	0.548
	Tot P	0.134	0.554*	−0.087	−0.006	−0.059	0.134	−0.086	0.857*	0.688**	0.222
	HS	0.196	−0.401	0.063	0.083	0.381	0.206	−0.288	−0.526	−0.755**	0.177

*P < 0.05; **P < 0.01.

The PI was ten times higher in the summer than in the winter–spring (Fig. 2F). In the summer, the PI showed a hump-shaped pattern from south to north, with the highest values in the northern Baltic Proper (stations 4–7). During the winter–spring relatively low values were observed. The PI correlated negatively with HSs and positively with salinity, temperature, Tot N and Tot P during the summer (Table I).

### Phytoplankton biomass and nutritional strategy

**Fig. 3.**
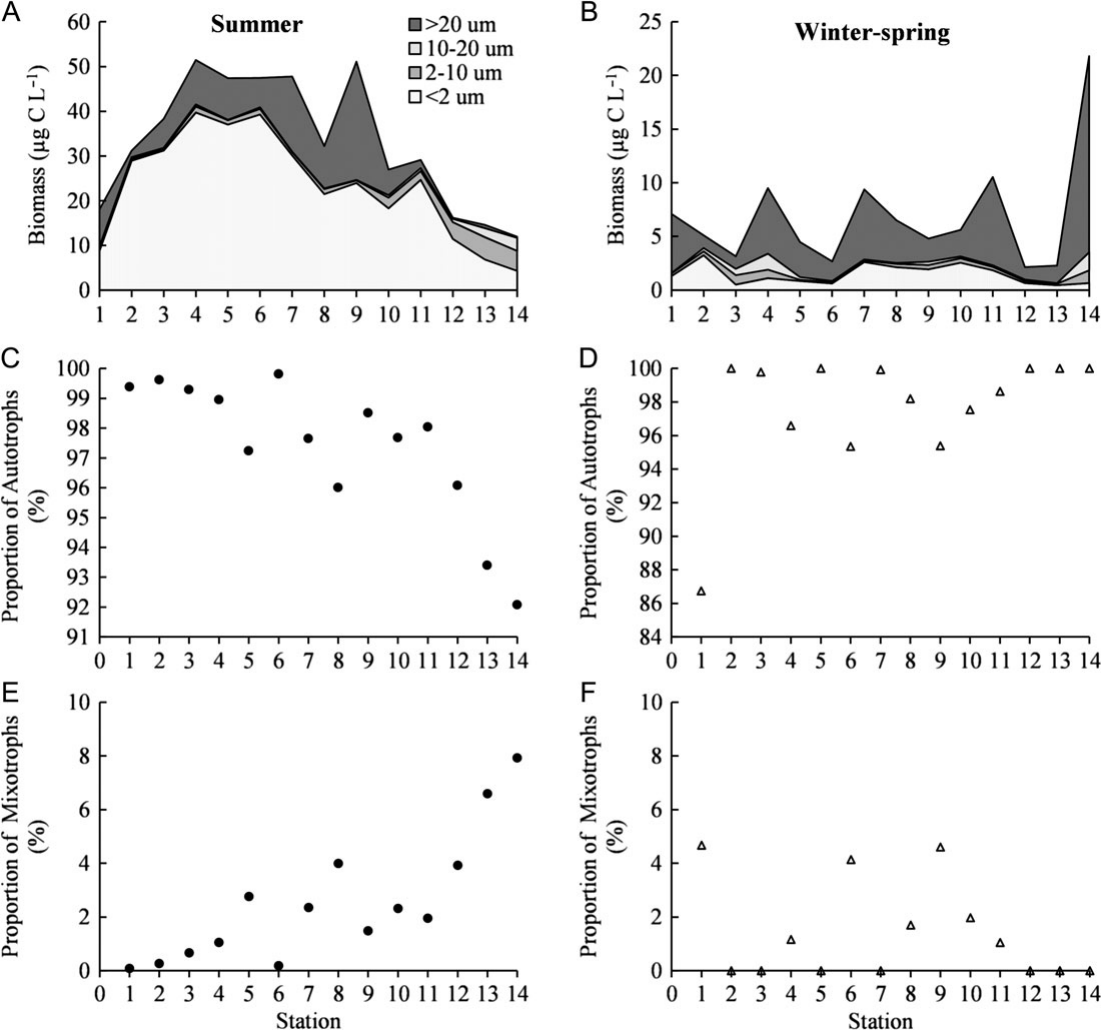
Phytoplankton biomass divided into four size groups (<2, 2–10, 10–20 and >20 µm) (**A**and**B)**, relative proportion (%) of autotrophs (**C **and**D**) and mixotrophs (**E **and** F**) during summer and winter–spring sampling periods.

During the summer the total phytoplankton biomass increased from the Kattegat station to the station located between the islands of Öland and Gotland (Fig. 3A). Northwards the phytoplankton biomass gradually decreased. The contribution of autotrophs was highest in the south and decreased gradually towards the north, while the proportion of mixotrophs increased northwards (Fig. 3C and E). Heterotrophs constituted an insignificant part of the biomass, and therefore they were not taken into account in the analysis. The total phytoplankton biomass was approximately five times higher in summer than in winter–spring (Fig. 3B) (Student's *t*-test: *t* = 7.634, df = 24, *P* < 0.001). In winter–spring, peaks of total pytoplankton biomass were observed in Kattegat, stations located close to islands (4, 7 and 11) and at the northernmost station in the Bothnian Bay (Fig. 3B). No specific trend in relation to nutritional strategy was observed during the winter–spring (Fig. 3D and F).

In the summer the total biomass was positively correlated with salinity, temperature, Tot N and Tot P, and negatively correlated with HSs (Table I). In winter–spring a positive correlation with Tot N was found (Table I).

### Size distribution and composition of phytoplankton

**Fig. 4.**
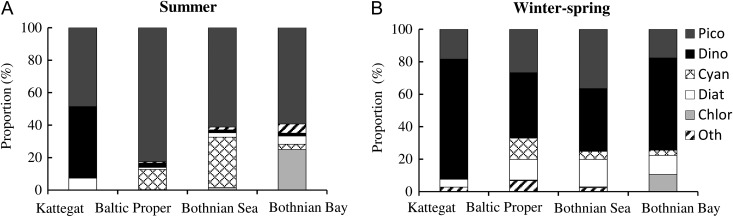
Relative biomass (%) of varying phytoplankton groups: Picocyanobacteria (Pico), Dinophyceae (Dino), Cyanophyceae (Cyan), Diatomophyceae (Diat), Chlorophyceae (Chlor) and Others (Oth) in different areas of the Baltic Sea

In the summer picoplankton (picocyanobacteria) was the dominant size group in all basins, while their relative contribution decreased gradually towards the north (Fig. 3A). Microplankton showed a similar spatial pattern, constituting ~25% of the biomass in the Baltic Proper and Bothnian Sea but only 4% in the Bothnian Bay. Ultraplankton and nanoplankton increased in importance towards the north and formed ~50–60% of the biomass in the northernmost stations in the Bothnian Bay (Fig. 3A). Picoplankton and microplankton were positively correlated with salinity, temperature, Tot N and Tot P and negatively with HSs (Table I). In contrast, the ultraplankton and nanoplankton showed a positive correlation with HSs and a negative correlation with other physicochemical variables. The second largest groups were Dinophyceae in the Kattegat, Cyanophyceae (colony-forming and/or filamentous cyanobacteria) in the Baltic Proper and Bothnian Sea, and Chlorophyceae in the Bothnian Bay (Fig. 4A).

During the winter–spring period, microplankton (Dinophyceae) dominated the phytoplankton community at all stations, forming on average ~50% of the biomass (Figs 3B and 4B). Picoplankton (picocyanobacteria) was the second most dominant fraction, and lowest concentrations were observed in the ice-covered Bothnian Bay. Ultraplankton and nanoplankton generally constituted <10% of the phytoplankton biomass. Positive correlations between picoplankton and Tot N and Tot P were observed, while microplankton only showed a positive correlation with Tot N (Table I). Picoplankton (Studen's *t*-test: *t* = 10.944, df = 24, *P* < 0.001) and ultraplankton biomass (Student's *t*-test: *t* = 4.065, df = 24, *P* < 0.001) showed significant differences between the seasons.

**Fig. 5.**
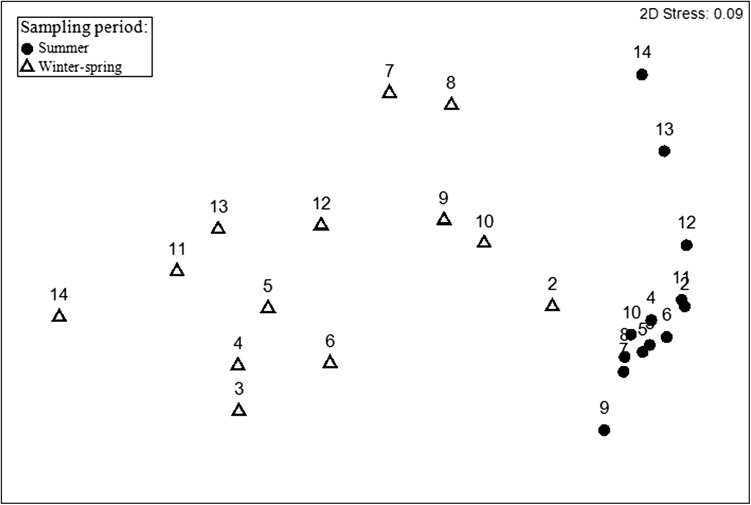
Non-multidimensional scaling ordination (NMDs) of phytoplankton community based on Bray–Curtis similarities of biomass during summer and winter–spring sampling periods. Numbers represent sampling stations.

NMDS ordination analysis showed that the phytoplankton community structure differed in the summer and the winter–spring (Fig. 5, ANOSIM global *R* = 0.746, *P* < 0.01). Furthermore, the phytoplankton community composition showed more differences in the south–north gradient during the winter–spring than during the summer, as indicated by the NMDS plot.

### Factors shaping the phytoplankton community

**Fig. 6.**
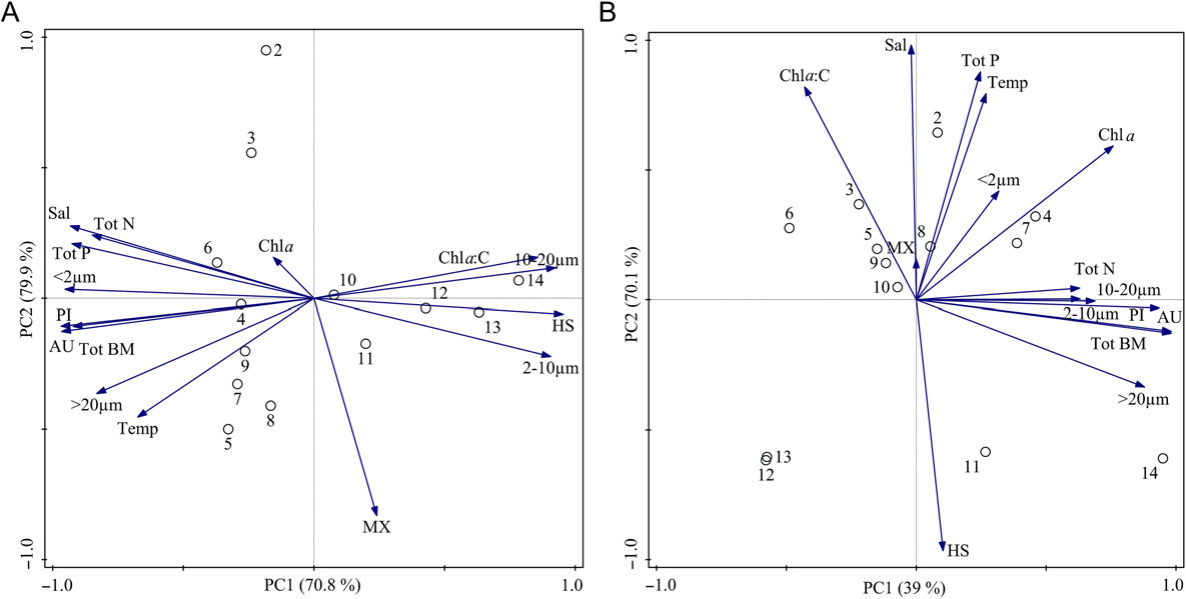
Principal component analysis (PCA) of the physicochemical variables and biological variables: salinity (Sal), temperature (Temp), total nitrogen (Tot N), total phosphorus (Tot P), humic substances (HS), size groups (< 2, 2–10, 10–20 and >20 µm) , mixotrophs (MX), autotrophs (AU), Chl *a*, Chl *a*:C, total biomass (Tot BM), photoprotective index (PI) during (A) summer and (B) winter–spring sampling periods.

In the PCA the first two axes explained 79.9% of the variance of the summer data, and 70.1% of the variance of the winter–spring data (Table II). In summer the highest principal component loadings were constituted by HSs (positive loading) and Tot P and salinity (negative loadings), while in winter–spring Tot N was the most important factor (positive loadings). The PCA indicated that in the summer HSs was a driver for the occurrence of ultra- and nanoplankton (partly consisting of mixotrophs), and high chlorophyll content in the phytoplankton cells, i.e. high Chl *a*:C ratio (Fig. 6A). This environmental condition was predominant at the most northerly stations. Autotrophic pico- and microplankton and the photoprotective pigments index were promoted by high Tot P and salinity, which were the prevalent environmental conditions at the more southerly stations. The PCA indicated that during winter–spring high Tot N concentration promoted e.g. total phytoplankton biomass and Chl *a* (Fig. 6B).

**Table II:** Results of principal component analysis

Season		Component	
		1	2
Summer	Salinity	−0.932	0.277
	Humic substances	0.954	−0.060
	Total phosphorus	−0.926	0.209
	Total nitrogen	−0.849	0.242
	Temperature	−0.674	−0.454
	Eigenvalues	0.7083	0.0909
	Cumulative,%	70.83	79.92
Winter–spring	Salinity	−0.020	0.981
	Humic substances	0.103	−0.966
	Total phosphorus	0.247	0.878
	Total nitrogen	0.629	0.044
	Temperature	0.269	0.794
	Eigenvalues	0.3898	0.3111
	Cumulative,%	38.98	70.09

## DISCUSSION

Our study indicates that ADOM, in addition to the more well-known factors (nitrogen, phosphorus and salinity), governs the large-scale patterns of phytoplankton nutritional strategy, size structure and pigment content in the Baltic Sea. During the summer, the relative proportion of autotrophic picoplankton was positively correlated with Tot P. This was somewhat unexpected because, applying ecological assumptions (Legendre and Rassoulzadegan, 1995), low-nutrient concentrations should lead to a dominance of smaller cells. Smaller phytoplankton cells have higher affinity for nutrient uptake and higher photosynthetic efficiencies than larger cells (Raven, 1998), but we did not find such responses at the ecosystem scale. Large autotrophic phytoplankton cells, >20 µm, showed a similar response as picoplankton. The decrease of the relative proportion of autotrophic pico- and microphytoplankton towards the north during the summer may thus have been caused by low-nutrient availability, deeper mixing and higher concentrations of HSs, which reduce the light availability in the water column (Kuosa, 1991). It is well-known that the northernmost part of the Baltic Sea (Bothnian Bay) is strongly phosphorus limited, while the Baltic Proper and the Swedish west coast generally are nitrogen-limited systems (Graneli *et al*., 1990; Andersson *et al*., 1996; Tamminen and Andersen, 2007).

Ultraplankton and nanoplankton were positively influenced by HSs during the summer. This indicates that these size groups have a different functional ecology compared with picocyanobacteria and larger phytoplankton. In fact, many of the species belonging to these groups are potentially mixotrophs, such as chrysophyceans (e.g. *Dinobryon* spp. and unidentified pigmented flagellates), dinophyceans and prymnesiophyceans (*Chrysochomulina* spp.). However, some purely autotrophic chlorophyceans (e.g. *Oocystis* spp.) also occurred in these size groups. Mixotrophs are likely to be promoted by HSs, as some of them feed on bacteria, which are capable of utilizing part of the ADOM (Andersson *et al*., 2015; Figueroa *et al*., 2016). These potential mixotrophs mainly occurred in the north, where the concentrations of HSs were higher and conditions more similar to humic lakes than the open sea. Our results are in agreement with studies in freshwater systems, where brown-water lakes have been shown to have higher abundances of mixotrophs compared with clear-water lakes (Bergström *et al*., 2003; Saad *et al*., 2013). This finding is less commonly recognized in marine systems, because usually only the coastal zone is markedly affected by ADOM. However, because of the high influence of river inflow in the north of the Baltic Sea, a strong structuring role of HSs was detected even in the offshore waters. Previous studies in the Baltic Sea have shown that mixotrophs are most abundant during the summer because of low-nutrient levels, which should promote phytoplankton with a diverse (i.e. mixotrophic) feeding mode and low salinity (Andersson *et al*., 1996; Hajdu *et al*., 1996; Dahl *et al*., 2005). However, our study indicates that low phosphorus and salinity and high humic concentrations selectively promoted this group in the summer.

This study shows that unicellular picocyanobacteria can be a dominant component of the phytoplankton community, forming 40–90% of the total phytoplankton biomass in the summer. The observed abundances of picocyanobacteria are comparable to previous studies in the Baltic Sea (Andersson *et al*., 1996; Hajdu *et al*., 2007). Stal *et al*. (1999) reported that 65% of the phytoplankton biomass in the Baltic Proper during late summer was composed of picoplankton, while the second most dominant group was nitrogen-fixing cyanobacteria (*Aphanizomenon* spp. and *Nodularia* spp.). Hajdu *et al*. (2007) showed that during the decline phase of cyanobacterial blooms in late summer, small diatoms, nanoflagellates, unicellular and colony-forming picocyanobacteria increase in abundance (Hajdu *et al*., 2007). From satellite image analysis we know that our summer sampling campaign was performed 1–2 weeks after the decline of extensive cyanobacterial blooms in the Baltic Proper (Hansson and Öberg, 2011), proving that the sampling was performed during the post-bloom period. In agreement with some earlier studies conducted during late phases of the summer bloom (Albertano *et al*., 1997; Stal *et al*., 1999), we found that unicellular picocyanobacteria and filamentous nitrogen-fixing cyanobacteria were the dominant phytoplankton groups. Thus, the general ecological relationship between nutrient availability and plankton size structure (Legendre and Rassoulzadegan, 1995) can be confounded by complex species interactions and successional timing during the year.

During winter–spring, autotrophic dinoflagellates dominated the phytoplankton community. This group has been reported to become more dominating during the spring bloom, possibly due to stronger vertical stratification induced by warmer seawater temperatures, which favor motile dinoflagellates over immotile diatoms (Wasmund and Uhlig, 2003). However, the present data-set does not directly support this theory, since dinoflagellates were the dominant group also in the coldest ice-covered basin, the Bothnian Bay.

Our study showed that the Chl *a*:C ratio and PI in the phytoplankton community was governed by a combination of different physicochemical variables. Thus phytoplankton inhabiting dynamic and changing environments, e.g. estuaries, may have a more varied pigment content than phytoplankton in the open sea. Chl *a* is commonly used as a proxy for total phytoplankton biomass in both monitoring and research programs (Andersson *et al*., 1996; HELCOM, 2013). However, our study shows that Chl *a* is a poor proxy for total phytoplankton biomass in the Baltic Sea because the Chl *a*:C ratio can vary by a factor of 10. We interpret the higher Chl *a*:C ratios in the northern Baltic during the summer as an effect of low light as a result of increased water color, which forced the phytoplankton to increase their chlorophyll *a* content to be able to capture light and sustain photosynthesis. Moreover, the increase in the Chl *a*:C ratios from south to north may also be caused by decreasing nitrogen limitation, as nitrogen is a major component of Chl *a*. On the other hand, it may appear strange that the Chl *a:*C ratio was highest where mixotrophic phytoplankton were most common. In some mixotrophs the chloroplasts are rudimentary and photosynthesis is used just as a survival mechanism when particulate food is scarce (e.g. Andersson *et al*., 1989). However, a large range of mixotrophic ecotypes occurs in aquatic systems, from almost purely autotrophic to almost purely heterotrophic (Jones, 2000). Furthermore, as the mixotrophs in our study formed at most only 10% of the total biomass, it is unlikely that they have a strong influence on the Chl *a*:C of the entire phytoplankton community.

The PI in the phytoplankton community was relatively high in the southern Baltic where concentrations of HSs were low. We interpret this to be a direct effect of a higher light intensity in the areas where the Tot P was also relatively high. In the northern region the concentrations of HSs were high, causing significant attenuation of the photosynthetically active radiation in the water column (Hoikkala *et al*., 2015). Previous studies have shown that the PI is governed by light conditions and that adaptation can occur in a very short period of time (Moisan *et al*., 1998), and that the photoprotective pigments increase in non-turbid clear waters and in surface water (Brunet *et al*., 1993; Riegman and Kraay, 2001). Certain cyanobacteria, e.g. *Synechococcus* spp., have some of the highest variations in the zeaxanthin:Chl *a* ratio under changing environmental conditions (Veldhuis and Kraay, 1990; Schlüter *et al*., 2000). Since picocyanobacteria dominated the phytoplankton community, they probably contributed to the observed geographical PI changes.

## CONCLUSIONS

In conclusion, we found that in the summer ADOM was one of the major factors governing the phytoplankton community, while Tot N played a major role in the winter–spring. The strong influence of ADOM in the northern region of the Baltic Sea (Bothnian Bay) favored ultraplankton and nanoplankton, which partly is formed by mixotrophic phytoplankton, capable of feeding on bacteria and other particulate matter. Furthermore, the brownish color of the ADOM causes shading in the seawater, which seemed to drive phytoplankton to increase their chlorophyll a content in the cells and to reduce the photoprotective pigments. Regional climate change projections indicate that within a 100-year period precipitation will increase in northern Europe, which will lead to increased river inflow of ADOM and a freshening of the Baltic Sea. We suggest that the observed changes in the phytoplankton community from north to south partly mirror future changes. The present northerly phytoplankton community structure in the future will transfer somewhat further south in the Baltic due to climate change.

## Supplementary Material

Supplementary Data

## References

[fbw081C1] Agawin N. S. R., Duarte C. M. and Agusti S. (2000) Nutrient and temperature control of the contribution of picoplankton to phytoplankton biomass and production. Limnol. Oceanogr., 45, 591–600.

[fbw081C2] Albertano P., Disomma D. and Capucci E. (1997) Cyanobacterial picoplankton from the Central Baltic Sea: cell size classification by image-analyzed fluorescence microscopy. J. Plankton Res., 19, 1405–1416.

[fbw081C3] Andersson A., Falk S., Samuelsson G. and Hagstrom A. (1989) Nutritional characteristics of a mixotrophic nanoflagellate, Ochromonas sp. Microb. Ecol., 17, 251–262. 24197284 10.1007/BF02012838

[fbw081C4] Andersson A., Hajdu S., Haecky P., Kuparinen J. and Wikner J (1996) Succession and growth limitation of phytoplankton in the Gulf of Bothnia (Baltic Sea). Mar. Biol., 126, 791–801.

[fbw081C5] Andersson A., Meier H. E. M., Ripszam M. et al (2015) Projected future climate change and Baltic Sea ecosystem management. Ambio, 44, 345–356. 26022318 10.1007/s13280-015-0654-8 PMC4447695

[fbw081C6] Behrenfeld M. J., Boss E., Siegel D. A. and Shea D. M. (2005) Carbon-based ocean productivity and phytoplankton physiology from space. Global Biogeochem. Cycles, 19, 1–14.

[fbw081C7] Bergström A. K., Jansson M., Drakare S. and Blomqvist P. (2003) Occurrence of mixotrophic flagellates in relation to bacterioplankton production, light regime and availability of inorganic nutrients in unproductive lakes with differing humic contents. Freshwater Biol., 48, 868–877.

[fbw081C8] Brunet C., Brylinski J. M. and Lemoine Y. (1993) In situ variations of the xanthophylls diatoxanthin and diadinoxanthin: photoadaptation and relationships with a hydrodynamical system in the eastern English Channel. Mar. Ecol. Prog. Ser., 102, 69–77.

[fbw081C9] Chisholm S. W. (1992) Phytoplankton size In: Falkowski P. G. and Woodhead A. D. Primary Productivity and Biogeochemical Cycles in the Sea. Plenum Press, New York, pp. 213–236.

[fbw081C10] Dahl E., Bagoien E., Edvardsen B. and Stenseth N. C. (2005) The dynamics of *Chrysochromulina* species in the Skagerrak in relation to environmental conditions. J. Sea Res., 54, 15–24.

[fbw081C11] Dahlgren K., Andersson A., Larsson U., Hajdu S. and Bamstedt U. (2010) Planktonic production and carbon transfer efficiency along a north-south gradient in the Baltic Sea. Mar. Ecol. Prog. Ser., 409, 77–94.

[fbw081C12] Dubinsky Z. and Stambler N. (2009) Photoacclimation processes in phytoplankton: mechanisms, consequences, and applications. Aquat. Microb. Ecol., 56, 163–176.

[fbw081C13] Edwards K. F., Thomas M. K., Klausmeier C. A. and Litchman E. (2015) Light and growth in marine phytoplankton: allometric, taxonomic, and environmental variation. Limnol. Oceanogr., 60, 540–552.

[fbw081C14] Erlandsson M., Buffam I., Folster J. et al (2008) Thirty-five years of synchrony in the organic matter concentrations of Swedish rivers explained by variation in flow and sulphate. Global Change Biol., 14, 1191–1198.

[fbw081C15] Evans C. D., Chapman P. J., Clark J. M. et al (2006) Alternative explanations for rising dissolved organic carbon export from organic soils. Global Change Biol., 12, 2044–2053.

[fbw081C16] Falkowski P. G. and Raven J. A. (2007) Aquatic Photosynthesis. Princeton University Press, Princeton.

[fbw081C17] Figueroa D., Rowe O. F., Paczkowska J. et al (2016) Allochthonous Carbon—a Major Driver of Bacterioplankton Production in the Subarctic Northern Baltic Sea. Microb. Ecol., 71, 789–801. 26677860 10.1007/s00248-015-0714-4 PMC4823372

[fbw081C18] Graneli E., Wallstrom K., Larsson U. et al (1990) Nutrient limitation of primary production in the Baltic Sea Area. Ambio, 19, 142–151.

[fbw081C19] Grasshoff K., Ehrhardt M. and Kremling K. (1983) Methods of Seawater Analysis, 2nd edn(Verlag Chemic, Weinheim, Germany.

[fbw081C20] Grover J. P. (1989) Influence of cell-shape and size on algal competitive ability. J. Phycol., 25, 402–405.

[fbw081C21] Hajdu S., Hoglander H. and Larsson U. (2007) Phytoplankton vertical distributions and composition in Baltic Sea cyanobacterial blooms. Harmful Algae, 6, 189–205.

[fbw081C22] Hajdu S., Larsson U. and Moestrup O. (1996) Seasonal dynamics of *Chrysochromulina* species (Prymnesiophyceae) in a coastal area and a nutrient-enriched inlet of the northern Baltic proper. Bot. Mar, 39, 281–295.

[fbw081C23] Hansson M., Öberg J. (2011) Cyanobacterial blooms in the Baltic Sea. HELCOM Baltic Sea Environment Fact Sheet 2011. (http://www.helcom.fi/Documents/Baltic%20sea%20trends/Environment%20fact%20sheets/BSEF_Cyanobacterial%20blooms%20in%20the%20Baltic%20Sea%202011.pdf)

[fbw081C24] Håkansson L. and Bryhn A. C. (2008) *Eutrophication in the Baltic Sea: Present Situation, Nutrient Transport Processes, Remedial Strategies*. Springer Science & Business media.

[fbw081C25] Havens K. E. (1998) Size structure and energetics in a plankton food web. Oikos, 81, 346–358.

[fbw081C26] HELCOM (2013) Climate change in the Baltic Sea area: HELCOM thematic assessment in 2013. In *Baltic Sea environment proceedings 137*.

[fbw081C27] Hoge F. E., Vodacek A. and Blough N. V. (1993) Inherent optical properties of the ocean: retrieval of the absorption coefficient of chromophoric dissolved organic matter from airborne laser spectral fluorescence measurements. Limnol. Oceanogr., 38, 1394–1402. 10.1364/AO.34.007032 21060564

[fbw081C28] Hoikkala L., Kortelainen P., Soinne H. and Kuosa H. (2015) Dissolved organic matter in the Baltic Sea. J. Mar. Syst., 142, 47–61.

[fbw081C29] Jones R. I. (2000) Mixotrophy in planktonic protists: an overview. Freshwat. Biol., 45, 219–226.

[fbw081C30] Kiørboe T. (1993) Turbulence, phytoplankton cell-size, and the structure of pelagic food webs. Adv. Mar. Biol., 29, 1–72.

[fbw081C31] Kirk J. T. O. (2011) Light and Photosynthesis in Aquatic Ecosystems. Cambridge University Press, Cambridge.

[fbw081C32] Kress N., Thingstad T. F., Pitta P., Psarra S., Tanaka T., Zohary T., Groom S., Herut B. et al (2005) Effect of P and N addition to oligotrophic eastern Mediterranean waters influenced by near-shore waters: a microcosm experiment. Deep-Sea Res. II, 52, 3054–3073.

[fbw081C33] Kuosa H. (1991) Picoplanktonic algae in the northern Baltic Sea: seasonal dynamics and flagellate grazing. Mar. Ecol. Prog. Ser., 73, 269–276.

[fbw081C34] Legendre L. and Rassoulzadegan F. (1995) Plankton and nutrient dynamics in marine waters. Ophelia, 41, 153–172.

[fbw081C35] MacIntyre H. L., Kana T. M., Anning T. and Geider R. J. (2002) Photoacclimation of photosynthesis irradiance response curves and photosynthetic pigments in microalgae and cyanobacteria. J. Phycol., 38, 17–38.

[fbw081C36] Meier H. E. M., Muller-Karulis B., Andersson H. C., Dieterich C., Eilola K., Gustafsson B. G., Hoglund A., Hordoir R. et al (2012) Impact of climate change on ecological quality indicators and biogeochemical fluxes in the Baltic Sea: a multi-model ensemble study. Ambio, 41, 558–573. 22926879 10.1007/s13280-012-0320-3 PMC3428484

[fbw081C37] Menden-Deuer S. and Lessard E. J. (2000) Carbon to volume relationships for dinoflagellates, diatoms, and other protist plankton. Limnol. Oceanogr., 45, 569–579.

[fbw081C38] Moisan T. A., Olaizola M. and Mitchell B. G. (1998) Xanthophyll cycling in *Phaeocystis antarctica*: changes in cellular fluorescence. Mar. Ecol. Prog. Ser., 169, 113–121.

[fbw081C39] Moreno D. V., Marrero J. P., Morales J., Garcia C. L., Ubeda M. G. V., Rueda M. J. and Llinas O. (2012) Phytoplankton functional community structure in Argentinian continental shelf determined by HPLC pigment signatures. Estuar. Coast. Shelf Sci., 100, 72–81.

[fbw081C40] Mousing E. A., Ellegaard M. and Richardson K. (2014) Global patterns in phytoplankton community size structure-evidence for a direct temperature effect. Mar. Ecol. Prog. Ser., 497, 25–38.

[fbw081C41] Olenina I., Hajdu S., Edler L. et al (2006) Biovolumes and size-classes of phytoplankton in the Baltic Sea. *HELCOM Baltic Sea Environment Proceedings*, 106. 144 pp.

[fbw081C42] Raven J. A. (1998) The twelfth Tansley Lecture. Small is beautiful: the picophytoplankton. Funct. Ecol., 12, 503–513.

[fbw081C43] Riegman R. and Kraay G. W. (2001) Phytoplankton community structure derived from HPLC analysis of pigments in the Faroe-Shetland Channel during summer 1999: the distribution of taxonomic groups in relation to physical/chemical conditions in the photic zone. J. Plankton Res., 23, 191–205.

[fbw081C44] Saad J. F., Schiaffino M. R., Vinocur A., O'farrell I., Tell G. and Izaguirre I. (2013) Microbial planktonic communities of freshwater environments from Tierra del Fuego: dominant trophic strategies in lakes with contrasting features. J. Plankton Res., 35, 1220–1233.

[fbw081C45] Schlüter L., Mohlenberg F., Havskum H. and Larsen S. (2000) The use of phytoplankton pigments for identifying and quantifying phytoplankton groups in coastal areas: testing the influence of light and nutrients on pigment/chlorophyll a ratios. Mar. Ecol. Prog. Ser., 192, 49–63.

[fbw081C46] Schlüter L., Mohlenberg F. and Kaas H. (2014) Temporal and spatial variability of phytoplankton monitored by a combination of monitoring buoys, pigment analysis and fast screening microscopy in the Fehmarn Belt Estuary. Environ. Monit. Assess., 186, 5167–5184. 24788839 10.1007/s10661-014-3767-9

[fbw081C47] Seekell D. A., Lapierre J. F., Ask J., Bergstrom A. K., Deininger A., Rodriguez P. and Karlsson J. (2015) The influence of dissolved organic carbon on primary production in northern lakes. Limnol. Oceanogr., 60, 1276–1285.

[fbw081C48] Spilling K., Ylostalo P., Simis S. and Seppala J. (2015) Interaction effects of light, temperature and nutrient limitations (N, P and Si) on growth, stoichiometry and photosynthetic parameters of the cold-water diatom *Chaetoceros wighamii*. Plos One, 10(5): e0126308 doi:10.1371/journal.pone.0126308. 25993327 10.1371/journal.pone.0126308 PMC4438981

[fbw081C49] Stal L. J., Staal M. and Villbrandt M. (1999) Nutrient control of cyanobacterial blooms in the Baltic Sea. Aquat. Microb. Ecol., 18, 165–173.

[fbw081C50] Tamminen T. and Andersen T. (2007) Seasonal phytoplankton nutrient limitation patterns as revealed by bioassays over Baltic Sea gradients of salinity and eutrophication. Mar. Ecol. Prog. Ser., 340, 121–138.

[fbw081C51] Thrane J. E., Hessen D. O. and Andersen T. (2014) The absorption of light in lakes: negative impact of dissolved organic carbon on primary productivity. Ecosystems, 17, 1040–1052.

[fbw081C52] Utermöhl H. (1958) Zur Vervollkommnung der quantitativen Phytoplankton-Methodik. Mitt. Int. Ver. Theor. Angew. Limnol., 9, 1–38.

[fbw081C53] Van Heukelem L. and Thomas C. S. (2001) Computer-assisted high-performance liquid chromatography method development with applications to the isolation and analysis of phytoplankton pigments. J. Chromatogr., 910, 31–49. 10.1016/s0378-4347(00)00603-4 11263574

[fbw081C54] Veldhuis M. J. W. and Kraay G. W. (1990) Vertical distribution and pigment composition of a picoplanktonic prochlorophyte in the subtropical North Atlantic: a combined study of HPLC-analysis of pigments and flow cytometry. Mar. Ecol. Prog. Ser., 68, 121–127.

[fbw081C55] Wasmund N. and Uhlig S. (2003) Phytoplankton trends in the Baltic Sea. ICES J. Mar. Sci., 60, 177–186.

[fbw081C56] Wedborg M., Skoog A. and Fogelqvist E. (1994) Organic carbon and humic substances in the Baltic Sea, Kattegatt and Skagerrak In: Sense N. and Miano T. M. Humic Substances in the Global Environment and Implications on Human Health. Elsevier Science, Amsterdam, pp. 917–924.

