# Supplementary material for: Allochthonous matter: an important factor shaping the phytoplankton community in the Baltic Sea
Source: J Plankton Res. 2016 Nov 26;39(1):23–34. doi: 10.1093/plankt/fbw081 (PMC5444108; doi:10.1093/plankt/fbw081)
Supplement: Supplementary Data [file fbw081_Supplementary_Data.zip › Supplementary_Material.docx]

**Supplementary Material:**

**Table S1.** Station coordinates and measured physicochemical variables, temperature (Temp), total phosphorus (Tot P), total nitrogen (Tot N) and humic substances (HS) during the summer and winter–spring samplings.

| Season | Station | Latitude | Longitude | Salinity | Temp (°C) | Tot P  (µmol L^-1^ ) | Tot N (µmol L^-1^) | HS  (µg L^-1^) |
| --- | --- | --- | --- | --- | --- | --- | --- | --- |
|  |  |  |  |  |  |  |  |  |
| Summer | 1 | 56°81´N | 12° 09´E | 18.3 | 18.5 | 0.4 | 19.6 | 5.0 |
|  | 2 | 55°18´N | 14° 29´E | 7.4 | 16.4 | 0.6 | 24.4 | 7.7 |
|  | 3 | 55°92´N | 16° 42´E | 6.6 | 16.7 | 0.5 | 23.3 | 7.9 |
|  | 4 | 57°13´N | 17° 58´E | 6.2 | 17.4 | 0.5 | 22.4 | 8.1 |
|  | 5 | 58°65´N | 19° 16´E | 6.3 | 18.2 | 0.5 | 23.9 | 7.8 |
|  | 6 | 59°08´N | 19° 67´E | 6.6 | 19.0 | 0.5 | 23.6 | 7.4 |
|  | 7 | 59°78´N | 19° 77´E | 5.4 | 17.7 | 0.5 | 21.8 | 8.6 |
|  | 8 | 60°91´N | 19° 27´E | 5.5 | 17.7 | 0.5 | 22.1 | 7.5 |
|  | 9 | 61°74´N | 19° 67´E | 5.4 | 17.4 | 0.4 | 20.6 | 7.6 |
|  | 10 | 62°51´N | 20° 03´E | 4.5 | 17.2 | 0.3 | 20.7 | 8.9 |
|  | 11 | 63°55´N | 20° 86´E | 3.2 | 16.6 | 0.3 | 16.0 | 11.7 |
|  | 12 | 64°23´N | 22° 30´E | 3.2 | 17.1 | 0.2 | 16.1 | 11.9 |
|  | 13 | 64°77´N | 23° 36´E | 3.0 | 16.8 | 0.2 | 15.6 | 12.4 |
|  | 14 | 65°16´N | 24° 63´E | 2.6 | 15.8 | 0.2 | 17.9 | 16.7 |
|  |  |  |  |  |  |  |  |  |
| **Winter –spring** | 1 | 56°81´N | 12° 09´E | 19.7 | 3.6 | 0.3 | 18.9 | 5.4 |
|  | 2 | 55°18´N | 14° 29´E | 7.9 | 2.6 | 0.8 | 13.4 | 6.1 |
|  | 3 | 55°92´N | 16° 42´E | 7.4 | 2.4 | 0.4 | 7.6 | 6.5 |
|  | 4 | 57°13´N | 17° 58´E | 7.2 | 2.9 | 0.5 | 10.9 | 6.5 |
|  | 5 | 58°65´N | 19° 16´E | 6.7 | 2.4 | 0.6 | 14.1 | 6.8 |
|  | 6 | 59°08´N | 19° 67´E | 6.8 | 2.6 | 0.4 | 9.1 | 6.8 |
|  | 7 | 59°78´N | 19° 77´E | 6.0 | 1.5 | 0.8 | 19.1 | 7.8 |
|  | 8 | 60°91´N | 19° 27´E | 5.7 | 1.1 | 0.4 | 12.6 | 7.2 |
|  | 9 | 61°74´N | 19° 67´E | 5.5 | 1.4 | 0.3 | 10.3 | 7.2 |
|  | 10 | 62°51´N | 20° 03´E | 5.6 | 1.1 | 0.3 | 9.8 | 6.8 |
|  | 11 | 63°55´N | 20° 86´E | 3.7 | 0.0 | 0.2 | 15.6 | 13.2 |
|  | 12 | 64°23´N | 22° 30´E | 3.1 | 0.2 | 0.1 | 9.3 | 13.8 |
|  | 13 | 64°77´N | 23° 36´E | 3.2 | 0.3 | 0.1 | 8.6 | 13.5 |
|  | 14 | 65°16´N | 24° 63´E | 3.1 | 1.8 | 0.2 | 13.1 | 15.6 |
|  |  |  |  |  |  |  |  |  |

**Table S2.** Pearson’s correlations between physicochemical variables during the summer and winter-spring samplings.

| Season | Variables | HS | Tot P | Tot N | Salinity | Temp |
| --- | --- | --- | --- | --- | --- | --- |
| Summer | HS  Tot P | -0.918** | -0.918** | -0.832**  0.912** | -0.938**  0.964** | -0.665*  0.477 |
|  | Tot N | -0.832** | 0.912** |  | 0.941** | 0.472 |
|  | Salinity | -0.938** | 0.964** | 0.941** |  | 0.532 |
|  | Temp | -0.665* | 0.477 | 0.472 | 0.532 |  |
| Winter**–**spring | HS  Tot P  Tot N | -0.795**  0.043 | -0.795**  0.469 | 0.043  0.469 | -0.969**  0.860**  0.027 | -0.723**  0.697**  -0.003 |
|  | Salinity | -0.969** | 0.860** | 0.027 |  | 0.790** |
|  | Temp | -0.723** | 0.697** | -0.003 | 0.790** |  |

*p<0.05;**p<0.01
